# Use of a Smartphone Self-assessment App for a Tobacco-Induced Disease (COPD, Cardiovascular Diseases, Cancer) Screening Strategy and to Encourage Smoking Cessation: Observational Study

**DOI:** 10.2196/19877

**Published:** 2022-02-23

**Authors:** Edouard Stavaux, François Goupil, Guillaume Barreau, Anne Lise Septans, Bertrand Dautzenberg, Armelle Foulet-Rogé, Norbert Padilla, Thierry Urban, Fabrice Denis

**Affiliations:** 1 Centre hospitalier universitaire de Nantes Nantes France; 2 Service de Pneumologie Centre Hospitalier du Mans Le Mans France; 3 Weprom Angers France; 4 Service de Pneumologie Hôpital de la Salpêtrière Paris France; 5 Service d'Anatomo-Pathologie Centre Hospitalier du Mans Le Mans France; 6 Centre de pathologie Maine Normandie Le Mans France; 7 Service de Pneumologie Centre Hospitalier Universitaire d'Angers Angers France; 8 Institut Inter-Regional de Cancérologie Jean Bernard Le Mans France

**Keywords:** smoking cessation, mobile health, self-assessment, lung cancer, early detection, tobacco-induced pathologies

## Abstract

**Background:**

Patient self-assessment via a mobile app detects actionable symptoms and has been shown to detect lung cancer relapses early, thereby lengthening survival.

**Objective:**

The purpose of this study was to assess the incidence of chief symptoms associated with the main tobacco-induced pathologies in both current and ex-smokers through a self-assessment smartphone app and to evaluate the app’s capacity to encourage users to quit smoking or reduce consumption, as well as its impact on early lung cancer stages at the time of diagnosis.

**Methods:**

Current and ex-smokers were recruited through an advertising campaign in Sarthe county (France) proposing the free download of a smartphone app. App users were asked to answer 13 questions related to symptoms associated with tobacco-induced diseases (chronic obstructive pulmonary disease [COPD], cardiovascular diseases, cancer). In the event of any positive answer, a message was displayed recommending the user to consult a physician. In addition, they were asked about smoking cessation intention before and after answering these 13 questions. Finally, incidence of stage 1 or 2 lung cancers diagnosed during the launch period of our application was evaluated by comparing data from various sources to those from the same period during the previous year.

**Results:**

Of the 5671 users who were eligible for evaluation, an alert was sent to the majority (4118/5671, 72.6%), with a higher incidence for current smokers (2833/3679, 77.0% vs 1298/1992, 65.2%; *P*<.001). The most frequent symptoms triggering the notifications were fatigue (2023/5671, 35.7%), cough (1658/5671, 29.2%), dyspnea (1502/5671, 26.5%), and persistent chest pain (1286/5671, 22.7%). Of the current smokers, 14.0% (515/3679) showed symptoms suggesting COPD, 15.5% (571/3679) showed symptoms suggesting stable angina, 12.4% (455/3679) probably had lower extremity artery disease, and 6.8% (249/3679) had possible cancer. Of the users, 36.5% (1343/3679) claimed that they thought about quitting smoking, and 48.7% (1795/3679) had thought about reducing their consumption. Surgery-eligible stage 1 and 2 lung cancer incidence was 24% (14/58) during the study period versus 9% (5/54) during the previous year in Sarthe county (*P*=.04), whereas it remained unchanged in the neighboring county of Maine-et-Loire.

**Conclusions:**

A majority of current and ex-smokers showed worrying symptoms, and the use of a self-assessment smartphone app may drive a majority of smokers toward the intention of smoking cessation or decreasing consumption. A randomized study should be performed to confirm this intention and to support the potential increase of symptomatic lung cancer detection at early, surgery-accessible stages.

**Trial Registration:**

ClinicalTrials.gov NCT04048954; https://www.clinicaltrials.gov/ct2/show/NCT04048954

## Introduction

Lung cancer is the most frequent cancer worldwide (2.09 million new cases in 2018) and the leading cause of death by cancer (1.76 million deaths in 2018) [1], whereas it is linked to an identifiable and avoidable risk factor in 90% of cases: tobacco. Moreover, 70% to 80% of lung cancers are diagnosed at a late and untreatable stage [2].

In addition, tobacco consumption may also result in other tumoral diseases (22% of deaths by cancer), cardiovascular diseases (CVD), and chronic pulmonary diseases such as chronic obstructive pulmonary disease (COPD), extremely frequent yet underevaluated pathologies (80% of the concerned patients ignore their status) related to tobacco consumption in 85% of cases. Nonspecific symptoms are commonly disregarded by patients [3] even though COPD was expected to be the third cause of morbidity and mortality worldwide in 2020. Evolution of COPD includes exacerbations, notably reducing patients’ quality of life, and a higher risk of lung cancer.

Yet, despite tobacco-cessation strategies ranging from awareness-raising campaigns to nicotine-replacement therapies, and—more recently—electronic cigarettes, long-term tobacco-cessation rates remain low [4].

New information and communication technology services in the form of mobile health (mHealth) have become a cornerstone in oncology. Various clinical studies have demonstrated longer survival in patients treated by chemotherapy [5] and earlier detection of lung cancer recurrence [6]. mHealth offers numerous advantages: Patients tend to better understand the stakes of their treatments and gain knowledge on sentinel symptoms, and doctors may react faster. In addition, patients treated for cancer do not feel more anxious with new access to medical information and, quite contrarily, show increased satisfaction regarding their care [7].

A study conducted in England in 2015 showed that reaching smokers with persistent cough through a simple poster advertising campaign led to the diagnosis of 9% more lung cancers over the campaign period and an increase in good-prognosis, surgery-eligible stage I cancer diagnosis by 3 percentage points. The tagline was “been coughing for 3 weeks or more? Tell your doctor” [8]. Current and ex-smokers often lack information regarding tobacco-related diseases [9] and frequently wait several months with symptoms before consulting a physician.

A dedicated self-assessment app provides regular analysis of symptoms in relation to tobacco consumption reported by patients on their smartphones and notifies them to consult their general practitioner (GP) in the event of suspicious symptoms. Knowledge about symptoms possibly indicating COPD, CVD, or lung diseases may help patients to reduce tobacco consumption or even quit smoking [10].

The purpose of this prospective study was to assess the incidence of the main symptoms associated with the leading tobacco-related diseases, evaluate the capacity of the app to encourage smokers and ex-smokers to quit smoking or reduce tobacco use, and evaluate its impact on the incidence of symptomatic surgery-eligible lung cancer.

## Methods

### Overview

Current and ex-smokers who quit smoking during the past 5 years were recruited via an advertising campaign on social media, newspapers, and public posters in the city of Le Mans and in Sarthe county (France). The campaign was held between June 3, 2019 and June 20, 2019. County GPs also received information on the app and were encouraged to propose it to their patients.

The self-assessment smartphone app was available for free download on Android and Apple app stores. After entering personal anonymous data (sex, age, tobacco consumption status, length and frequency of tobacco consumption), current smokers were asked whether they considered quitting or did not wish to at all. Both current and ex-smokers were then requested to answer 13 simple questions linked to symptoms (chest pain, chest tightness, cough, dyspnea, unusual tiredness, lower limb claudication, hematuria, hemoptysis, unintentional weight loss, dysphagia, dysphonia, palpable subcutaneous nodule, need to expectorate) possibly corresponding with the following diseases: cancer (lung, head and neck, esophagus, kidney, bladder), COPD, lower extremity arterial disease (LEAD), and angina. In the event of a positive answer to any of the mentioned points, a notification was sent recommending the user to consult his or her GP about the suspicious symptoms.

Symptoms were selected by a board of experts according to specific semiology of each disease [6,11].

In addition, once the questionnaire was filled out, current smokers were asked whether they considered reducing tobacco consumption or quitting. In addition, a toll-free telephone number was provided for patient counseling on smoking cessation therapy.

### Inclusion and Exclusion Criteria

For the purpose of this study, participants were included based on the following criteria: aged 16 years or older, being a current or former smoker (cessation within the past 5 years), informed consent on data use.

Exclusion criteria were a lack of consent and nonuse of the app.

### Ethical Approval

The study was approved by the French National Health Data Institute, which reviews ethical issues in human research, data confidentiality, and safety.

We focused on the diagnosis of cancer in the 2 counties as data were available through the registries of thoracic surgical oncology, unlike information regarding COPD or arteritis. In addition, surgery remains the main curative treatment for lung cancer.

### Statistical Analysis

We first assessed the number of users presenting with symptoms. We then evaluated the impact on smoking cessation intention after using the app and compared the rate of symptomatic lung cancer eligible for surgery in Sarthe county and neighboring Maine-et-Loire during the app roll-out period with the same period in 2018. Analysis of new cases started 12 weeks after launching the app to allow time for required examinations (eg, endoscopy, computed tomography [CT] scan, positron emission tomography scan). This evaluation was conducted on the basis of private and public pathology laboratories’ records in both Sarthe and Maine-et-Loire counties, thus providing 100% of documented lung cancer cases. Records from multidisciplinary team thoracic oncology meetings in both counties were analyzed to obtain the number of symptomatic lung cancers eligible for surgery.

Fisher and Mac-Neymar tests were used for descriptive analyses, with *P*<.05 considered to be statistically significant.

## Results

### Recruited Population

**Figure 1:**
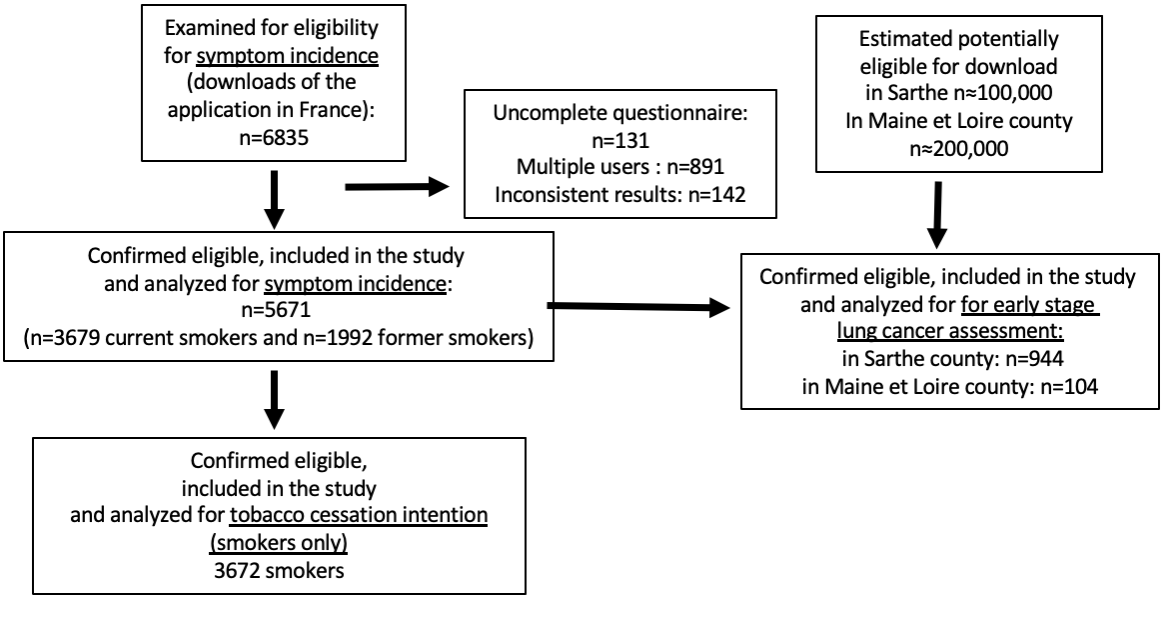
CONSORT (Consolidated Standards of Reporting Trials) diagram.

The app was downloaded 6835 times between June 3, 2019 and December 31, 2019, and 5671 users were eligible. Among them, 3679 (64.9%) were current smokers, and 1992 (35.1%) were ex-smokers. Median age was 39 years, 57.8% (3276/5671) were men, and 42.2% (2395/5671) were women. Regarding smoking history, 3425 (3425/5671, 60.4%) had a 20 pack-year smoking history, and 4450 (4450/5671, 78.5%) had been smoking for at least 20 years. The app was downloaded by 944 users in Sarthe county and 104 in neighboring Maine-et-Loire (Figure 1). In step 1, the baseline questions were asked of current smokers only (n=3679). These questions asked about smoking cessation (answers = Yes/No), to which 627 answered “Yes” to “You don’t want to stop smoking?” and 3052 answered “Yes” to “Or are you hesitating to stop?” In step 2, 13 questions about symptoms were asked of the entire study population (n=5671). In Step 3, after completing the symptom questionnaire, the users were asked “At the end of the first use of Smokecheck, have you considered continuing your current consumption, without a reduction in consumption (Yes: 531/3672, 14.4%), reducing your consumption (Yes: 1796/3672, 49.0%), or total cessation of tobacco use (Yes: 1345/3672, 36.6%)?”

### Notifications

A notification was sent to a majority of users (4118/5671, 72.6%). Current smokers were notified significantly more often than ex-smokers (2833/3679, 77.0% vs 1298/1992, 65.2%; *P*<.001). The most frequently detected symptoms were unusual tiredness (2023/5671, 35.7%), persistent cough (1658/5671, 29.2%), dyspnea (1502/5671, 26.5%), and persistent chest pain (1286/5671, 22.7%; Table 1).

**Table 1:** Symptoms self-assessed on a smartphone app by current or former smokers who stopped smoking within the past 5 years.

Symptoms that triggered a notification to the user^a^	Current smokers (n=3679), n (%)	Former smokers (n=1992), n (%)	*P* value
Have you noticed persistent, unusual tiredness that has lasted for 3 weeks?	1401 (38.1)	622 (31.2)	<.001
Have you been coughing for 3 weeks or more?	1269 (34.5)	389 (19.5)	<.001
Have you had, for 3 weeks or more, unusual shortness of breath when walking on flat terrain compared with someone of the same age?	1092 (29.7)	410 (20.6)	<.001
Do you have unusual shoulder pain or chest pain that has been persistent for 3 weeks or more?	870 (23.6)	416 (20.9)	.01
In the last 3 past weeks, have you had any pain or a squeezing sensation in your chest during effort?	571 (16)	222 (11.1)	<.001
In the previous few weeks, have you had pain in 1 or both legs while walking that has caused you to stop walking?	455 (12.4)	205 (10.3)	.02
Do you currently have a change in your voice that has lasted 3 weeks or more?	380 (10.3)	161 (8.1)	.01
Have you unintentionally lost 3 kg or more in the last 3 months?	325 (8.8)	123 (6.2)	<.001
Have you recently noticed an unusual lump under your skin?	303 (8.2)	155 (7.8)	.57
Have you had persistent difficulties swallowing food for 3 weeks or more?	126 (3.4)	77 (3.9)	.41
In the last 3 weeks, have you observed the presence of blood in your sputum?	82 (2.2)	49 (2.5)	.52
In the past 3 weeks, have you observed blood in your urine?	43 (1.2)	18 (0.9)	.42

^a^In the event of a positive answer to any of the questions, a notification was sent recommending the user to consult his or her general practitioner about the suspicious symptoms.

### Symptoms and Smoking Duration

We observed that smokers with a longer smoking history were more frequently notified.

Indeed, 78.0% (3213/4118) of the patients who received a notification had a smoking history of over 20 years, while 22.0% (905/4118) had less than 20 years of smoking history (*P*<.001).

### Number of Symptoms

On average, users who received a notification had 1.93 symptoms. Current smokers had 2.13 symptoms, whereas former smokers had 1.57 symptoms (*P*<.001).

### Tobacco-Induced Diseases

Several symptoms, isolated or in association, may suggest tobacco-induced diseases accessible to specific screening.

Associated symptoms that may be indicators of COPD, such as a persistent cough for 3 weeks with dyspnea or a need to expectorate, was reported by 14% (515/3679) of current smokers as well as 7.5% (150/1992) of ex-smokers (*P*<.001).

Lower-limb pain suggestive of LEAD was found in 12.4% (455/3679) of notified current smokers and 10.3% (205/1992) of notified ex-smokers (*P*=.02).

Of current smokers who received a notification, 15.5% (571/3679) presented with symptoms suspicious of stable angina, compared with 11.1% (222/1992) of ex-smokers (*P*<.001).

Of current smokers, 6.8% (249/3679) were notified for symptoms suggesting pulmonary, urinary, or head-and-neck cancer, such as hemoptysis, hematuria, and unintentional weight loss associated with chest pain or dysphagia, as were 5.9% (117/1992) of former smokers (*P*=.20)

### More Specific Symptoms

Asthenia associated with another symptom may suggest tobacco-induced complications, but it cannot be considered as specifically linked to tobacco consumption when isolated. Provided that the app also recorded notifications to patients who had only checked this symptom in the app, we removed these data and then noticed a lower number of notifications, amounting to 3784, representing 66.7% (3784/5671 vs 4118/5671, 72.6%), with 71.1% (2615/3679) of current smokers and 58.0% (1157/1992) of ex-smokers notified.

### Cessation Intention

After using the app, 36.5% (1343/3679) of users considered quitting smoking, 48.7% (1795/3679) considered reducing their consumption, and 14.4% (530/3679) did not plan any change.

Before answering the 13-item questionnaire, 627 of 3679 current smokers (17.0%) had declared that they did not wish to quit smoking. However, 5.7% (36/627) changed their mind and declared they wanted to quit after completing the questionnaire (*P*<.001; Table 2).

Among the 3052 smokers who hesitated to quit before answering the 13 questions, 42.8% (1306/3052) then declared their intention to quit, and 48.5% (1480/3052) declared an intent to reduce their consumption.

After completing the questionnaire, 1490 of the 1949 smokers who had declared that they wanted to reduce their consumption had received a notification (76.4%) and so did 1368 of the 1772 (77.2%) who declared that they wanted to quit.

**Table 2:** Consideration of smoking cessation as reported by 3679 current smokers before the symptom questionnaire intervention and subsequent intention for smoking reduction or cessation after completing the symptom questionnaire in the app.

Status of current smokers at baseline		Smoking intention after app use, n (%)				*P* value^a^
		No smoking change	Smoking reduction	Smoking cessation		
Would not consider cessation (n=627)		269 (42.9)	316 (50.4)	36 (5.7)	<.001	
**Alert status for those who would not consider cessation**						
	Alert received (n=470)	201 (42.8)	235 (50.0)	29 (6.2)	-^b^	
	No alert received (n=157)	68 (43.3)	81 (51.6)	7 (4.5)	-	
Would consider cessation (n=3052)		261 (8.6)	1479 (48.5)	1307 (42.8)	<.001	
**Alert status for those who would consider cessation**						
	Alert received (n=2361)	202 (8.6)	1138 (48.2)	1019 (43.2)	-	
	No alert received (n=689)	59 (8.6)	341 (49.5)	288 (41.8)	-	

^a^Assessed using the Mac-Neymar test.

^b^Comparisons not conducted.

### Incidence of Symptomatic Lung Cancers Eligible for Surgery

#### In Sarthe County (944 Downloads)

Between June 21, 2018 and August 21, 2018, 54 new cases of lung cancer were diagnosed, whereas 58 new cases were diagnosed over the same period in 2019 (ie, 12 weeks after launching the app in Sarthe county).

**Figure 2:**
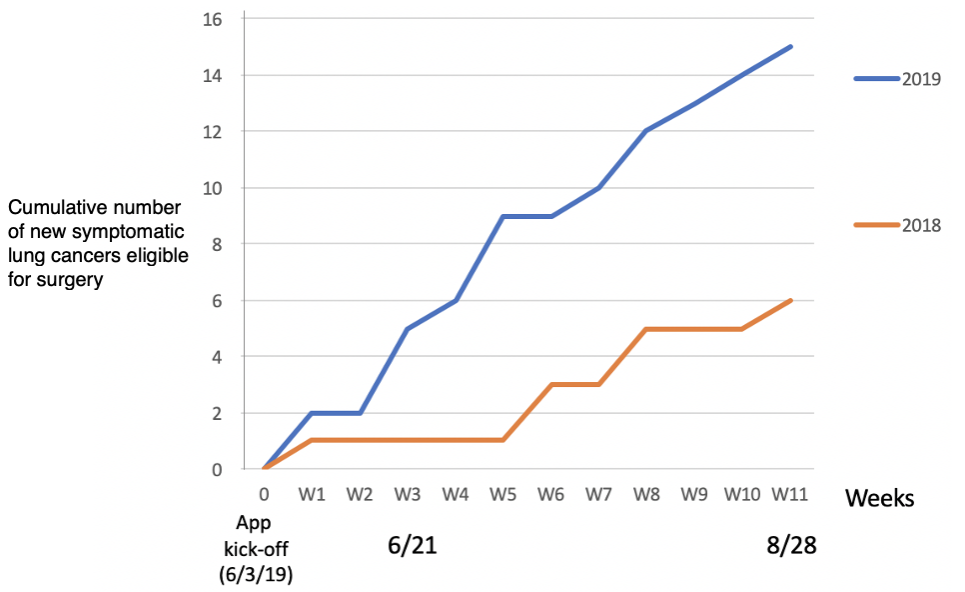
Cumulative number of new symptomatic lung cancers eligible for surgery during the 12 weeks after the application kick-off in 2019 (assessed between June 21, 2019 and August 28, 2019) and during the same period without application availability in Sarthe in 2018.

The number of symptomatic lung cancers eligible for surgery increased from 9% (5/54) during that period in 2018 to 24% (14/58) over the same period in 2019 (*P*=.004; Figure 2, Table 3).

**Table 3:** Patients with symptomatic lung cancer characteristics between June 21, 2018 and August 28, 2018 and after the app kick-off in Sarthe county during the same period in 2019 (kick-off on June 3, 2019).

Year and patient number		Age (years)	Symptom(s)	TNM^a^	Stage	Histology	Surgery	Performed	pTNM^b^
**2018**									
	Patient 1	64	Persistent cough	T2N1M0	2	ADK^c^	Yes	Yes	pT2pN1
	Patient 2	70	Chest pain	T2N0M0	1	ADK	Yes	Yes	pT1pN0
	Patient 3	64	Dyspnea	T1N0M0	1	ADK	Yes	Yes	pT1pN0
	Patient 4	64	Cough	T2N0M0	1	ADK	Yes	Yes	pT3pN2
	Patient 5	69	Cough asthenia	T2N1M0	2	SCC^d^	Yes	Yes	pT3pN1
**2019**									
	Patient 1	59	Persistent cough	T2N0M0	1	ADK	Yes	Yes	pT2pN2
	Patient 2	60	Chest pain	T1N0M0	1	K sarc^e^	Yes	Yes	pT3pN2
	Patient 3	88	Persistent cough	T1N0M0	1	ADK	Yes	Yes	pT1pN0
	Patient 4	51	Cough and hemoptysis	T1N0M0	1	Carcinoid	Yes	Yes	pT1pN2
	Patient 5	51	Persistent cough and asthenia	T3N0M0	2	SCC	Yes	Yes	pT2pN1
	Patient 6	54	Persistent cough and asthenia	T2N0M0	1	SCC	Yes	No	No surgery
	Patient 7	64	Dyspnea	T2N0M0	1	ADK	Yes	Yes	pT2pN1
	Patient 8	53	Lump under skin	T1N0M0	1	ADK	Yes	Yes	pT3pN0
	Patient 9	59	Persistent cough	T1N0M0	1	ADK	Yes	Yes	pT1pN1
	Patient 10	29	Persistent cough	T1N0M0	1	Carcinoid	Yes	Yes	pT1pN0
	Patient 11	66	Chest pain and weight loss	T3N0M0	2	SCC	Yes	Yes	pT4pN1
	Patient 12	75	Persistent cough and weight loss	T1N0M0	1	ADK	Yes	Yes	pT2pN0
	Patient 13^f^	46	Pain in one leg	T1N0M0	1	ADK	Yes	Yes	pT1pN0
	Patient 14^f^	58	Pain in one leg	T1N0M0	1	ADK	Yes	Yes	pT1pN0

^a^TNM: staging classification that describes the tumor (T), node (N), and metastasis (M) categories.

^b^pTNM: pathological tumor-node-metastasis.

^c^ADK: adenocarcinoma.

^d^SCC: squamous cell carcinoma.

^e^K sarc: sarcomatoid carcinoma.

^f^Experienced leg claudication that led to a visit.

#### In Neighboring Maine-et-Loire County (104 Downloads)

The number of symptomatic lung cancers eligible for surgery was stable between 2018 (10/74, 14%) and 2019 (12/78, 15%) in this county, where the app was not deployed (*P*=.82).

## Discussion

### Principal Findings

#### Representative Population and Important Use of the App Despite an Advertising Campaign Limited in Time and Space

To the extent of our knowledge, this is the first prospective real-life study with a self-assessment app aiming to detect symptoms of smokers and ex-smokers in order to encourage them to quit smoking and to consult their physician for early detection of tobacco-induced diseases, such as lung cancer. Despite a local campaign in a single county (Sarthe) over a limited 2-week period, the app was downloaded nearly 7000 times, 80% of which occurred in other counties. Social networks and word of mouth among users’ families certainly spread the use of the app further. This strong participation allowed it to quickly reach a high number of users with clinical features similar to those of smokers in other studies. Indeed, the median age of smokers and ex-smokers, who had quit within the past 5 years, was 39 years, with 42.2% (2395/5671) of our study population as women. Hajek et al [12] randomized e-cigarettes and nicotine replacement therapy, with a median age of 41 years and 48% women.

#### Frequency of Symptoms and Diseases

A majority of users received a notification (72.6%), especially among current smokers (77.0% vs 64.6% of ex-smokers).

A significant number of smokers presented with symptoms or associations of symptoms suggesting tobacco-induced diseases: 14.0% with COPD, 15.5% with stable angina, 12.4% with potential LEAD, and 6.8% with potential cancer. Number and incidence of symptoms indicating these diseases appeared to be lower after recent cessation (<5 years), which contributes to showing that smoking cessation can help to prevent them.

#### Smoking Cessation Intention

After using the app, a significant number of current smokers declared their intention to reduce their tobacco consumption (1795/3679, 48.7%), and 36.5% (1343/3679) declared their intention to quit altogether.

Among those who declared an intention to reduce consumption after filling out the questionnaire, 76.4% received a notification through the app, and 77.2% of those who stated that they wanted to quit were notified.

Even though the questionnaire only collected declarations of intent, which cannot be easily verified in practice, triggered notifications brought to light some concerns, which seemed to prompt smokers to reduce consumption or even quit smoking. This behavior change elicited by raised awareness about tobacco-induced symptoms is a new personalized approach, as smokers are directly and objectively facing the abnormality of such symptoms. These can lead to smoking cessation, as has been observed among 42.8% of the smokers who claimed to be hesitant about quitting (representing 83% of all smokers in this study).

#### Cancer Screening

In the United States, the US Preventive Services Task Force established guidelines for lung cancer screening. Patients have to be over the age of 55 years, have a 30-pack year history of smoking, and be a current or former smoker [13], but our app is not a “lung cancer screening” tool as per US specificities but actually aims to refer users to their physician in case of suspicious symptoms. It therefore returns an alert, independent of the user’s age or smoking history. The launch of the app in June 2019 was linked to a significant increase in the number of symptomatic surgery-eligible lung cancers within the 12 weeks following the start of the advertising campaign and the no-cost availability of the app in Sarthe county where it was downloaded 944 times. Over the same period in 2018, only 9% of lung cancers diagnosed based on symptoms were eligible for surgery. In the neighboring Maine-et-Loire county, where the app was only downloaded 104 times, the number of symptomatic cancers diagnosed at an operable stage had not increased significantly (14% between June 21, 2018 and August 28, 2018; 15% between the same dates in 2019). These figures suggest a positive correlation between the availability of the app (as well as its download) and the increase in detections of symptomatic lung cancers eligible for surgery.

### Limitations

This study had several limitations. First, there was no control group. Second, the app is a self-assessment app in which information is self-reported, rather than a patient-reported outcomes app that reports data to physicians. Thus, data were left unverified, whether regarding symptoms or smoking cessation intentions. Several symptoms triggering notifications are not very specific of tobacco-induced diseases when isolated and thus raise the number of user alerts. By removing isolated asthenia from all notifications (n=334), we reached a rate of notification of 66.7% (vs 72.6%), and by removing isolated subcutaneous nodules (n=79), we reached 65.3%. These 2 symptoms, when recorded as isolated symptoms, could be removed from further versions of the app, in order to be more specific in detecting tobacco-induced complications. This may also prevent the difficult absorption by physicians of the numerous additional consultations expected after nationwide coverage.

In order to evaluate the impact of the app on the detection of tobacco-induced diseases, a questionnaire could have been sent to users a few weeks after receiving the alert encouraging to consult a doctor. The app was not designed with this characteristic, which could be grounds for further study. We decided to focus on lung cancer and chose the eligibility for curative surgery as our primary outcome.

The increase in the incidence of surgery-eligible cancers was not assessed through direct contact with users, as this study was based on health data requiring full anonymity. Evaluation was indirectly conducted, by triangulating oncology and pathology records, which gather nearly 100% of cases in the concerned counties (Sarthe and Maine-et-Loire), as well as data from app downloads. This result would have been more relevant if a direct link between the use of the app and cancer diagnosis had been established, but the chosen methodology could not allow it. GPs’ awareness-raising activities and the advertising campaign itself also may have contributed to the early detection of these cancers.

### Generalizability

Among smokers and ex-smokers, symptoms related to tobacco-induced diseases are frequent. The app is a self-assessment mHealth tool that could encourage GP consults whenever suspicious symptoms appear, setting up strong prevention dynamics in total safety as opposed to some use of e-cigarettes. Physicians could also implement earlier treatments against diseases such as COPD, which is a significant risk factor for cancer and the third cause of mortality in France, as well as coronary and other arterial diseases, early treatment of which also provide major benefits. The early detection and management of these diseases may lead to reduced cancer incidence and support the preventive role of this app against lung cancer. Finally, this app may be a relevant tool to prompt symptomatic smokers to schedule a thoracic CT, with the hope to foster participation in this modality of cancer screening, whose benefit on survival rates was proven despite weak participation in real life [14,15].

This exploratory study warrants a follow-up randomized study to evaluate the impact of this tool on early lung cancer screening.

## Abbreviations

COPD: chronic obstructive pulmonary disease
CT: computed tomography
CVD: cardiovascular disease
GP: general practitioner
LEAD: lower extremity artery disease
mHealth: mobile health

## References

[1] (2018). Cancer. World Health Organization.

[2] (2013). Tumeur maligne, affection maligne du tissu lymphatique ou hématopoïétique. Haute Autorite de Sante.

[3] (2019). Connaissance de la bronchopneumopathie chronique obstructive (BPCO) en France : Baromètre santé 2017. Sante publique France.

[4] Gomajee R, El-Khoury F, Goldberg M, Zins M, Lemogne C, Wiernik E, Lequy-Flahault E, Romanello L, Kousignian I, Melchior M (2019). Association between electronic cigarette use and smoking reduction in France. JAMA Intern Med.

[5] Basch E, Deal AM, Dueck AC, Scher HI, Kris MG, Hudis C, Schrag D (2017). Overall survival results of a trial assessing patient-reported outcomes for symptom monitoring during routine cancer treatment. JAMA.

[6] Denis F, Basch E, Septans A, Bennouna J, Urban T, Dueck AC, Letellier C (2019). Two-year survival comparing web-based symptom monitoring vs routine surveillance following treatment for lung cancer. JAMA.

[7] Gravis G, Protière C, Eisinger F, Boher JM, Tarpin C, Coso D, Cappiello M, Camerlo J, Genre D, Viens P (2011). Full access to medical records does not modify anxiety in cancer patients: results of a randomized study. Cancer.

[8] Moffat J, Bentley A, Ironmonger L, Boughey A, Radford G, Duffy S (2015). The impact of national cancer awareness campaigns for bowel and lung cancer symptoms on sociodemographic inequalities in immediate key symptom awareness and GP attendances. Br J Cancer.

[9] (2021). Global briefing: symptom awareness and attitudes to lung cancer Findings from a global study. Global Lung Cancer Coalition.

[10] Ironmonger L, Ohuma E, Ormiston-Smith N, Gildea C, Thomson CS, Peake MD (2015). An evaluation of the impact of large-scale interventions to raise public awareness of a lung cancer symptom. Br J Cancer.

[11] Guillevin L (2011). Sémiologie médicale, 2ème édition.

[12] Hajek P, Phillips-Waller A, Przulj D, Pesola F, Myers Smith K, Bisal N, Li J, Parrott S, Sasieni P, Dawkins L, Ross L, Goniewicz M, Wu Q, McRobbie HJ (2019). A randomized trial of e-cigarettes versus nicotine-replacement therapy. N Engl J Med.

[13] (2021). Final Recommendation Statement: Lung Cancer: Screening. U.S. Preventive Services Task Force.

[14] Tanoue L (2012). Reduced lung-cancer mortality with low-dose computed tomographic screening. Yearbook of Medicine.

[15] de Koning HJ, van der Aalst CM, de Jong PA, Scholten ET, Nackaerts K, Heuvelmans MA, Lammers JJ, Weenink C, Yousaf-Khan U, Horeweg N, van ’t Westeinde S, Prokop M, Mali WP, Mohamed Hoesein FA, van Ooijen PM, Aerts JG, den Bakker MA, Thunnissen E, Verschakelen J, Vliegenthart R, Walter JE, ten Haaf K, Groen HJ, Oudkerk M (2020). Reduced lung-cancer mortality with volume CT screening in a randomized trial. N Engl J Med.

